# Health workforce remuneration: comparing wage levels, ranking, and dispersion of 16 occupational groups in 20 countries

**DOI:** 10.1186/1478-4491-11-11

**Published:** 2013-02-28

**Authors:** Kea Tijdens, Daniel H de Vries, Stephanie Steinmetz

**Affiliations:** 1 Amsterdam Institute for Advanced Labor Studies (AIAS), University of Amsterdam, Amsterdam, The Netherlands; 2 Department of Sociology & Anthropology, Center for Social Science and Global Health, University of Amsterdam, Amsterdam, The Netherlands; 3 Department of Sociology & Anthropology, University of Amsterdam, Amsterdam, The Netherlands; 4 Department of Sociology, Erasmus University, Rotterdam, the Netherlands

**Keywords:** Health workforce composition, Remuneration, Wages, Survey data, Occupational groups, Ranking, Dispersion, Donuses

## Abstract

**Background:**

This article represents the first attempt to explore remuneration in Human Resources for Health (HRH), comparing wage levels, ranking and dispersion of 16 HRH occupational groups in 20 countries (Argentina, Belarus, Belgium, Brazil, Chile, Colombia, the Czech Republic, Finland, Germany, India, Mexico, the Netherlands, Poland, Russian Federation, Republic of South Africa (RSA), Spain, Sweden, Ukraine, United Kingdom (UK), and United States of America (USA)). The main aim is to examine to what extent the wage rankings, standardized wage levels, and wage dispersion are similar between the 16 occupational groups and across the selected countries and what factors can be shown to be related to the differences that emerge.

**Method:**

The pooled data from the continuous, worldwide, multilingual *WageIndicator* web survey between 2008 and 2011 (for selected HRH occupations, *n*=49,687) have been aggregated into a data file with median or mean remuneration values for 300 occupation/country cells. Hourly wages are expressed in standardized US Dollars (USD), all controlled for purchasing power parity (PPP) and indexed to 2011 levels.

**Results:**

The wage ranking of 16 HRH occupational groups is fairly similar across countries. Overall Medical Doctors have the highest and Personal Care Workers the lowest median wages. Wage levels of Nursing & Midwifery Professionals vary largely. Health Care Managers have lower earnings than Medical Doctors in all except six of the 20 countries. The largest wage differences are found for the Medical Doctors earning 20 times less in Ukraine than in the US, and the Personal Care Workers, who earn nine times less in the Ukraine than in the Netherlands. No support is found for the assumption that the ratio across the highest and lowest earning HRH occupations is similar between countries: it varies from 2.0 in Sweden to 9.7 in Brazil. Moreover, an increase in the percentage of women in an occupation has a large downward effect on its wage rank.

**Conclusions:**

This article breaks new ground by investigating for the first time the wage levels, ranking, and dispersion of occupational groups in the HRH workforce across countries. The explorative findings illustrate that the assumption of similarity in cross-country wage ranking holds, but that wage dispersion and wage levels are not similar. These findings might contribute to the policies for health workforce composition and the planning of healthcare provisions.

## Abstract (es)

**Abstract in Spanish:**

**Antecedentes**

Este artículo representa el primer intento de explorar la remuneración de los Recursos Humanos para la Salud (RHS), la comparación de los niveles salariales, clasificación, y dispersión de los 16 grupos de trabajo en 20 países (Argentina, Belarús, Bélgica, Brasil, Chile, Colombia, la República Checa, Finlandia, Alemania, India, México, los Países Bajos, Polonia, la Federación de Rusia, la República de Sudáfrica, España, Suecia, Ucrania, el Reino Unido, y los Estados Unidos de América (EEUU)). El objetivo principal es analizar en qué medida los rankings de salarios, niveles estandarizados de salarios, y la dispersión salarial es similar entre los 16 grupos de trabajo y en los países seleccionados y qué factores se puede demostrar relacionados con las diferencias que surjan.

**Métodos**

Los datos agrupados de la continua encuesta mundial, multilingüe *WageIndicator* por Internet entre 2008 y 2011 (para determinadas ocupaciones de RHS, *n*=49,687) se han agrupado en un archivo de datos con los valores de la remuneración promedio o media para 300 células de ocupación/país. Los salarios por hora se expresan en estandarizados dólares estadounidenses (USD), todos controlados por la paridad del poder adquisitivo (PPP) e indexados a los niveles de 2011.

**Resultados**

El ranking salarial de 16 grupos ocupacionales de RHS es bastante similar en todos los países. En general los médicos tienen los más altos y los trabajadores de atención personal los más bajos salarios medios. Los niveles salariales varían en gran medida para los profesionales de Enfermería y Partería. Los gerentes de salud tienen menores ingresos que los médicos en todos los países examinados excepto en seis. Las mayores diferencias salariales se encuentran entre los médicos, que ganan 20 veces menos en Ucrania que en los EEUU, y los trabajadores de cuidado personal, que ganan nueve veces menos en Ucrania que en los Países Bajos. No se ha encontrado apoyo a que exista la supuesta relación entre los índices de salarios más altos y más bajos de las profesiones RHS entre estos países, sino que varía de 2,0 a 9,7 en Suecia en Brasil. Además, cuando existe un alto porcentaje de mujeres en ciertas profesiones hay una baja en su rango salarial.

**Conclusiones**

Este artículo abre nuevas fronteras al investigar por primera vez los niveles salariales, la clasificación, y la dispersión de los grupos ocupacionales de la fuerza de trabajo SAR entre ciertos países. Los hallazgos exploratorios muestran que la hipótesis de la similitud en el ranking de salarios entre países se mantiene, son sólo la dispersión de los salarios y los niveles salariales que no son similares. Estos hallazgos podrían contribuir a las políticas para la composición del personal sanitario y a la planificación de las medidas de servicios de la salud.

## Abstract (de)

**Abstract in german:**

**Hintergrund**

Dieser Artikel stellt den ersten Versuch dar, einen Einblick in die Vergütung (Lohnniveau, -ranking und –verteilung) von 16 Berufsgruppen aus dem Gesundheitsbereich (HRH) in 20 Ländern (Argentinien, Belarus, Belgien, Brasilien, Chile, Kolumbien, Tschechien, Finnland, Deutschland, Indien, Mexiko, den Niederlanden, Polen, Russische Föderation, Republik Südafrika (RSA), Spanien, Schweden, Ukraine, Vereinigtes Königreich (UK), und Vereinigte Staaten von Amerika (USA)) zu erhalten. Hauptziel ist es, zu prüfen, inwieweit sich die 16 Berufsgruppen und ausgewählten Länder im Hinblick auf das standardisierte Lohnniveau, das Lohn-Ranking und die Lohnverteilung ähnlich sind, und welche Faktoren für die beobachteten Unterschiede verantwortlich gemacht werden können.

**Methode**

Die gepoolten Daten der kontinuierlichen, weltweiten und mehrsprachigen *WageIndicator-*Onlineumfrage zwischen 2008 und 2011 (für ausgewählte HRH Berufe N = 49.687) wurden zu einer Datei aggregiert, die die Vergütung für 300 Beruf/Land-Zellen in Median- oder Mittelwert-Werten angibt. Die Stundenlöhne wurden in standardisierten US-Dollar (USD) unter Kontrolle der Kaufkraftparität (PPP) angegeben und sind auf dem Niveau von 2011 indexiert.

**Ergebnisse**

Die Analyse zeigt, dass das Lohnranking der16 HRH Berufsgruppen zwischen den Ländern vergleichbar ist. In allen Ländern haben Ärzte die höchste und persönliche Betreuungskräfte die niedrigsten mittleren Löhne. Es zeigt sich jedoch auch, dass große Länderunterschiede hinsichtlich des Lohnniveaus von professionellen Krankenpflegern und Geburtshelfern bestehen. Mit Ausnahme von 6 der 20 Länder erzielen Manager im Gesundheitswesen geringere Erträge als Ärzte. Die größten Lohnunterschiede sind für die Berufsgruppe der Ärzte zu beobachten: ukrainische Ärzte verdienen 20-Mal weniger als ihre US-amerikanischen Kollegen. Ein ähnliches Ergebnis zeigt sich auch für individuelle Pflegekräfte in der Ukraine, die 9-mal weniger verdienen als die gleiche Berufsgruppe in den Niederlanden. Die Analyse widerlegt die Annahme, dass das Verhältnis zwischen den höchsten und niedrigsten HRH-Berufen über die Länder hinweg vergleichbar ist: Der Wert variiert zwischen 2.0 in Schweden und 9.7 in Brasilien. Darüber hinaus zeigt sich,dass ein ansteigender Prozentsatz von Frauen zu einer starken Herabstufung im Lohnrang der betreffenden Berufsgruppe führt.

**Schlussfolgerungen**

Dieser Artikel beschreibt zum ersten Mal die Ähnlichkeiten und Unterschiede bezüglich des Lohnniveaus, des Lohnrankings und der Lohnverteilung von 16 HRH-Berufsgruppen für 20 ausgewählte Länder. Er untersuch des Weiteren mögliche Einflussfaktoren. Die explorativen Befunde bestätigen die Annahme eines ähnlichen/vergleichbaren Lohnrankings in den untersuchten Ländern, während das Lohnniveau und die Lohnverteilung zwischen den Ländern nicht vergleichbar sind. Diese Erkenntnisse könnten dazu beitragen, Richtlinien und Politiken für die Zusammensetzung des Gesundheitspersonals sowie die Planung der Gesundheitsversorgung zu entwickeln.

## Background

Wages are central to healthcare. Wages set the frame for health workforce composition. Wage information is needed for planning healthcare provisions. Wages affect job and life satisfaction, employment, and working conditions as well as retention, attrition, or migration of professionals within and across countries. Wages are commonly perceived as a key factor affecting job satisfaction and migration of healthcare professionals within and across countries [1-4]. Wages are a main component of labor costs, which in turn determine local or regional health workforce composition decisions. A major problem preventing progress on insight into the relative importance of wage information in health workforce strengthening is the lack of detailed information about the wide range of health workers’ occupations [5]. Typically, international databases employ high levels of occupational aggregation and are insufficiently standardized in their classifications to allow for cross-country comparability [6]. For example, while the October Inquiry and the Occupational Wages (OWW) database of the International Labour Organization (ILO) is an important resource, only seven occupations are included for the health sector (general physician, dentist, professional nurse, auxiliary nurse, physiotherapist, medical X-ray technician, and ambulance driver). Another major source, the Luxembourg Income and Employment Study, has surveyed 30 countries over the past decades, yet lacks sufficient specificity - as most labor force surveys do - by not providing further detail than a two-digit coding of ILO’s International Standard Classification of Occupations (ISCO). Recent research on wages for a number of European countries concludes that no cross-country comparable wage data are available for the occupational groups in the Human Resources for Health (HRH) workforce, and that one has to rely on a few national studies with incomparable wage data and incomparable occupations [7]. At the country level, a small diversity of HRH sources is available including population censuses and surveys, facility assessments, and routine administrative records. However, most available data sources have shortcomings [8,9]. A recent inventory of the requirements for a Human Resources Information System (HRIS) identified pay roll data as one of the nine components needed for data collection, but showed that these data are hard to collect [10]. Only in 10% of the 63 countries under review were these data available, with the result that the availability of pay roll data was ranked as the second-lowest component.

As a result of this absence of comparable wage data, few studies have investigated wage levels and wage distribution in the health workforce across countries [6,11]. Preliminary analysis has suggested that salary differentials between source and destination countries are too high to curb migration [11]. Using data on 42 countries from both the OECD Health Data 2005 and OWW database for a comparison of wages of general physicians and professional nurses only, Dräger *et al*. found that there is an enormous gap in wages for health workers between rich and poor countries [6]. Moreover, health workers tend to be paid less than equivalent professionals - or at least teachers and engineers - in low-income countries. Wages, they suggest, are great incentives for health workers to migrate, posing challenges for the development of strategies to retain them in poor countries. At the same time, an increasingly complex remuneration landscape in destination countries reveals the development of different task profiles and related certification requirements - a proxy for relative wage ranking for distinct occupations [12,13]. The differences and the complexity of wage structures between various countries are of potential key influence to workforce migratory patterns, workforce composition decisions, the national settings of healthcare provision, the wage setting processes, and professional credentialing (certification, licensing, and professional registration).

Given the absence of pay roll data in many countries and the subsequent problem of harmonizing pay roll data across countries, this research uses cross-country comparable survey data on wages. The analysis aims to detail and compare the wage structure in the HRH workforce across countries. It tests to what extent countries are similar with respect to ranking of wage levels and the wage dispersion across occupations. Moreover, it examines whether workforce composition characteristics of the occupations, such as age, gender, and employment status, influence the national wage ranking and wage levels. The following research objectives are investigated:

1. To what extent are the HRH occupational groups across countries similar with respect to the wage levels, the wage ranking, the wage dispersion, and the incidence of bonuses? And to what extent are the countries similar across HRH occupational groups?

2. To what extent do the gender, age, and employment status composition as well as the bonuses within the occupation/country cells show a relationship with their wage rankings and levels?

## Methods

### Data

The data used in this study stem from the self-administered *WageIndicator* questionnaire, which is posted continuously at all national *WageIndicator* websites (http://www.wageindicator.org). This dataset is particularly suited to our research objectives, because it offers a variety of detailed and comparable wage and occupation variables across a range of countries. Due to the fundamental data limitations highlighted above, the *WageIndicator* dataset offers a unique window to study in detail wage levels, ranking, and dispersion in HRH occupational groups. The first website of *WageIndicator* started in the Netherlands in 2001, and is operational today in 75 countries in five continents, receiving millions of visitors. The websites consist of job-related content, labor law and minimum wage information, VIP wages, and a free Salary Check presenting average wages for occupations based on the web survey data. Web traffic is high due to coalitions with media groups with a strong Internet presence, search engine optimization, web-marketing, publicity, mobile applications, and answering visitors’ email. The websites are consulted by employees, self-employed persons, students, job seekers, individuals with a job on the side, and similarly for their annual performance talks, job mobility decisions, occupational choices, or other reasons. In return for the free information provided, web visitors are invited to voluntarily complete a questionnaire with a lottery prize incentive. Between 1% and 5% of the visitors do so. Since the start of the survey, more than 1 million visitors to the website have provided valid information about their weekly, monthly, or annual wages. The survey consists of two parts which each take approximately 10 minutes to complete. In countries with poor Internet access the questionnaire is restricted to part one only. The questionnaire is comparable across countries. It is in the national language(s), adapted to country peculiarities, and asks questions about a wide range of subjects, including basic sociodemographic characteristics, wages, occupations, and other work-related topics (see Additional file 1: Stylized questionnaire; the codebook is downloadable [14]; for academic research the data are available for free from the IZA, Bonn, Germany, http://idsc.iza.org/?page=27&stid=1025).

With respect to the quality of the dataset, its volunteer nature is a challenge. In the scientific community, the increasing use of web surveys has triggered a heated debate about their quality and reliability for scientific use [15,16]. On the one hand, web surveys offer a number of advantages, such as cost benefits, fast and continuous data collection, and the potential to reach respondents across national borders. On the other hand, they have been criticized for the absence of an adequate sampling frame and in respect to the related question of whether the collected data are representative of the population of interest. The subpopulation with Internet access, the subpopulation visiting the web survey’s website, and the subpopulation deciding to complete the survey are quite specific. Therefore different calibration techniques (post-stratification weighting and propensity score adjustment) have been considered with the aim to deal with the described problem. Two approaches were used: improving the quality of web survey estimates and adjusting the biased web sample to the population under consideration [17,18]. In the case of the *WageIndicator* data, a study of six countries (Germany, the Netherlands, Spain, USA, Argentina, and Brazil) shows that in 2006 most web samples deviated to some extent from the reference samples with regard to the common variables of age, gender, and education [19,20]. These findings are similar to findings from previous research [21,22]. As a consequence, a simple proportional weighting procedure has been applied, adjusting the annual gender and age distributions of *WageIndicator* to those of ILO’s global Economically Active Population Estimates and Projections (EAPEP, 6th edition) [23]. More advanced calibration methods could not be applied, due to a fundamental lack of more recent, suitable, and comparable reference surveys, in particular for low-income countries. The simple weights, however, indicate that in almost all countries the labor force aged 40 years and over is under-represented in our dataset, for women more so than for men. Explanations may be related to their relatively higher linguistic and computer illiteracy rates. In India, women aged 40 years and over were the most under-represented with a weighting factor of 14.2, followed by Mexico (4.5) and Belarus (3.2). For the remaining gender-age categories, however, our weights fluctuated between 0.5 and 2.0. Given the non-representative nature of the data, and despite these weights, the results should be considered explorative rather than representative for the HRH workforces in the 20 countries. For the research reported in this article, the described simple proportional within country-weighting procedure was used. The sample is not weighted across countries, because we use occupation/country cells as the unit of analysis, as explained in the next section.

### Selecting health sector occupations and countries

In the *WageIndicator* web survey, respondents self-identify their occupation by means of a three-step search tree allowing them to navigate easily through a multilingual database with 1,700 occupational titles, including a large number of health sector occupations. All occupational titles are coded according to the ILO’s recently updated standard occupational classification (ISCO-08) [24]. The health sector occupations in the database were selected and subsequently clustered into 20 health sector occupational groups (hereafter called HRH occupations), following the classifications in the Communicable Disease Global Atlas for Human Resources for Health of the WHO [25] and the ILO’s definition of health sector occupational units [26]. However, we kept a number of more detailed occupational categories in order to gain additional insight in the wage structure in the HRH workforce across countries. In two cases, the definition was ambiguous and led to the exclusion of occupations related to pharmaceutical production and job holders in the Health Care Administration & Operations occupation (non-managerial), who were not employed in the healthcare sector (NACE2.0 codes 86, 87, 88). Administrative and non-health-related skills are core to the latter occupation and can be used in any industry (see Additional file 2: Mapping the selected *WageIndicator* occupations into the 20 HRH occupations and their ISCO-08 codes).

For this study, the *WageIndicator* survey data from 2008 to 2011 have been pooled to obtain sufficient observations for the HRH occupations. We trust pooling the data, because it seems unlikely that the wage ranking across the HRH occupations changes rapidly over a short period. Moreover, the wages have been corrected across years, as will be described in the next section. The number of observations per country varies largely, because the response is related to the number of web visitors, which in turn depends on the start date of the website, cooperation with media partners, the size of the country, the number of competing websites, Internet access rates in the country, and so on. Given the aim of wage comparisons across HRH occupations and across countries, we set the commonly used threshold of at least five observations with wage information in an occupation/country cell. This led to an exclusion of four HRH occupations (Traditional & Complementary Medicine (Associate) Professionals, Paramedical Practitioners, Veterinary Professionals, and Optometrists and Ophthalmic Opticians) of the initial list of 20. In the analysis, 16 HRH occupations could be compared across 20 countries, but for 20 occupation/country cells we had fewer than five observations. Hence, the analyses are based on 300 occupation/country cells (16* 20–20). The 20 countries stem from four continents, namely one country from Africa (RSA), six from the Americas (Argentina, Brazil, Chile, Colombia, Mexico, USA), one from Asia (India) and 12 from Europe (Belarus, Belgium, Czech Republic, Finland, Germany, the Netherlands, Poland, Russian Federation, Spain, Sweden, Ukraine, UK). Given the applied selection, the weighted *WageIndicator* data have 49,687 observations. For the occupations, the number of observations ranged from 370 for Dentists to 9,432 for the Health Care Administration & Operations occupations (non-managerial). For the 20 countries, the number of observations ranged from 299 for Sweden to 13,509 for Germany.

In this study, the units of analysis are occupation/country cells, thus occupations within countries. This implies that we do not control for the number of job holders across the occupations within a country and we also do not control for the size of the HRH workforces across countries. The aggregate data file of 300 occupation/country cells comprises the medians or means of the variables of interest, namely wages and bonuses, as well as age, gender, and employment status, as will be explained hereafter (see Additional file 3: The aggregate data file of the 16 occupations * 20 country cells). The survey has information about second and third jobs. However, because fewer than 5% of respondents reported on this, this information was not included in the analysis.

### Defining hourly wages

The *WageIndicator* web survey asks respondents in dependent and self-employed labor relations about their earnings in detail. Both groups are routed differently through the survey because the questions on wages and income are different [14]. The employees are asked if they are paid per month or per week, whichever is most common in the country of survey. If the answer is ‘no’, the next question asks them to select the pay period. In the few countries where it is deemed necessary, a question asks about the currency in which the wage is paid. The next question asks if respondents know their gross and net wage. Depending on the answer, questions ask for the last gross and/or net wage. Here, a probe suggests respondents to specifically include bonuses, if these were received in the last wage. A question then presents a list of bonuses that may have been included in the last wage, ranging from shift and commuting allowances to tips and performance bonuses. These questions are set to the default ‘no’. If ‘yes’ is selected, a question pops up asking for the amount of the bonus. The self-employed respondent group receives questions about their gross annual income before taxation, followed by a question whether this income was earned in 12 months or less, and if less, in how many months. The page with the bonus questions is not asked of the self-employed.

For the employees, the gross hourly wages were computed from their weekly hours, their pay period, and their gross wages. Weekly hours were derived from the contractual weekly hours for employees with agreed working hours in their employment contract and from the usual weekly working hours for all other categories. The pay period was self-reported in the survey. When only net wages were reported, the gross wages have been computed-based on the annual country average between gross and net wages in 10 wage brackets. The reported bonuses were categorized as regular or annual, for example, seniority bonus *versus* end-of-year bonus, and as fixed *versus* variable, for example, holiday allowance *versus* bonus from profits. The fixed regular bonuses were included in the hourly wages. The fixed annual bonuses were included proportionally in the hourly wages. The variable bonuses were excluded from the hourly wage computation, because in some years and some cases they greatly affected the wages. Employer-provided services, such as healthcare insurance and non-monetary remuneration, food vouchers, or laptops were not included in the hourly wages, because the monetary value of such services was not asked in the survey. Allowances for expenses, such as commuting allowances, were also excluded. Overtime bonuses were excluded from the computation of the hourly wages, because overtime hours only exist for employees with contractual working hours, which were the basis of the hourly wage computation. For the self-employed, their annual income was divided by their weekly working hours, multiplied by the usual national working weeks per year and controlled for the months worked. For convenience, we use the term hourly wages also for the self-employed.

Once the gross hourly wages in national currencies were computed, they were standardized into US Dollars (USD) using the PPP projections from the World Bank Database. The PPP theory uses the long-term equilibrium exchange rate of two currencies to equalize their purchasing power for a given basket of goods. In the data cleaning, odd values in the reported gross and/or net wages were set to missing. The standardized hourly wages lower than 1 PPP standardized USD or higher than 400 PPP standardized USD were considered outliers. Note that the common 1% rule to identify outliers was not applied, because of the continuous nature of the survey. Given that datasets are prepared quarterly, this threshold for outliers would have varied too much across the quarters. To compare the standardized gross hourly wages over the survey years, the 2008 wages were augmented with the ratio of the national PPP-2011/PPP-2008, and similarly for 2009 and 2010. Thus, all wages were indexed to the 2011 level. In the remainder of this article, the term standardized USD gross hourly wages will be used to refer to the PPP standardized wages in USD, indexed to the 2011 level. Because of the skewed distribution of wages, we preferred the median of the hourly wages over the mean in our aggregate data file with the 300 occupation/country cells.

## Results

### Wage structures in HRH occupations

**Figure 1:**
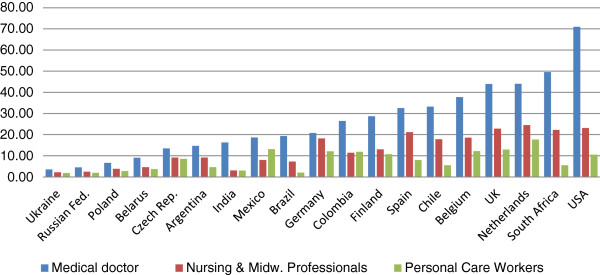
Median gross hourly wages in standardized USD in three HRH occupations in 20 countries.

Research objective 1 aimed to investigate the extent to which HRH occupational groups across countries are similar with respect to the wage levels, the wage ranking, the wage dispersion, and the incidence of bonuses. Thus, how do the occupational wage structures compare internationally? And to what extent are the countries similar across HRH occupational groups? Before turning to the overall picture, the gross median standardized wages for three occupations, namely for the groups of Medical Doctors, Nursing & Midwifery Professionals, and Personal Care Workers in Health Services are shown as an example (Figure 1). The largest wage differences for the group of Medical Doctors are between Ukraine and USA. A Ukrainian doctor earns 20 times less than a US doctor, using PPP standardized wages. The Nursing & Midwifery Professionals occupational group exhibits the same pattern, though the differences are smaller. In the Ukraine, this group earns 11 times less than their Dutch counterparts, who have the highest earnings. When it comes to the group of Personal Care Workers, the pattern is similar. Again, care workers in Ukraine have the lowest earnings, earning nine times less than care workers in the Netherlands.

The wage levels of the 16 HRH occupational groups are shown in Table 1. Across the 20 countries, Medical Doctors are paid the highest wages and Personal Care Workers the lowest ones. The maximum wages earned in a country are highest for Dentists and again lowest for Personal Care Workers. Table 2 shows a transposed picture, revealing the wage levels of the joint HRH occupations in the 20 countries. The overall HRH wages are highest in the Netherlands, UK, and USA and lowest in Poland, the Russian Federation, and Ukraine (Table 2, column 2). The highest wages in a HRH occupation are paid in Belgium and the USA, and the lowest wages are found in Ukraine and the Russian Federation.

**Table 1:** Mean of the median hourly occupational wages in the 20 countries, and its standard deviation, minimum, and maximum (wages in standardized USD, indexed to the 2011 level), mean of the occupational wage rank across 20 countries, its standard deviation, minimum, and maximum (1=lowest rank, 16 = highest rank), number of countries in which the occupation ranks highest and ranks lowest, and number of countries with valid observations

	**Standardized hourly wages (USD)**				**Wage rank (1=lowest, 16 = highest)**				**Countries with highest rank ( *n *)**	**Countries with lowest rank ( *n *)**	**Countries with valid observations ( *n *)**
	**Mean wage**	**SD mean wage**	**Min. wage**	**Max. wage**	**Mean rank**	**SD rank**	**Min. rank**	**Max. rank**			
Medical Doctors	26.02	17.64	3.55	71.01	14.3	2.2	9.0	16.0	7	0	19
Dentists	25.80	24.65	2.95	72.85	13.1	3.3	7.4	16.0	4	0	12
Pharmacists	19.99	14.61	2.45	48.58	12.4	3.7	1.0	16.0	1	1	17
Health Researchers & Educators	16.76	8.47	4.67	35.85	12.7	2.9	4.2	16.0	4	0	20
Health Care Managers	15.86	6.85	3.76	28.92	12.8	2.4	8.5	16.0	4	0	20
Envir. & Occ. Health Professionals	14.02	7.86	2.74	27.67	11.1	2.5	5.0	14.9	0	0	17
Nursing & Midwifery Professionals	13.06	7.84	2.20	24.47	8.0	3.6	3.0	14.9	0	0	20
Physiotherapists	12.47	6.88	3.84	25.63	8.0	3.6	1.0	13.0	0	1	18
Other Health Professionals	12.15	6.12	3.62	22.64	8.0	2.9	4.0	14.9	0	0	20
Health Informatics Technicians	11.77	6.07	4.01	24.70	7.8	3.7	2.0	15.0	0	0	20
Community Health Workers	11.57	6.51	2.02	23.47	6.0	3.5	2.2	13.0	0	0	20
Nurses & Midwifery Associate Professionals	11.41	7.58	1.90	23.42	4.9	3.6	1.0	13.0	0	3	18
Medical and Pharmaceutical Technicians	11.37	5.42	3.84	20.37	7.5	3.1	2.1	14.0	0	0	20
Other Health Associate Professionals	10.39	5.31	2.82	19.00	4.9	2.1	1.0	11.0	0	1	20
Health Care Administration & Operations	9.94	5.18	2.90	19.02	4.6	2.9	1.0	12.5	0	1	20
Personal Care Workers in Health Services	7.82	4.79	1.83	17.65	2.3	2.9	1.0	10.6	0	13	19
Total	14.06	10.55	1.83	72.85	8.5	4.6	1.0	16.0	20	20	300

Source: Data from the *WageIndicator* survey 2008–2011 (weighted within countries), aggregated for 16 HRH occupations in 20 countries (*n*=300 occupation/country cells, which are not weighted across countries for the size of the national population or the national HRH workforce).

**Table 2:** Mean of the median hourly national wages of the 16 HRH occupations, and its standard deviation, minimum, and maximum (wages in standardized USD, indexed to the 2011 level), max/min ratio, correlation coefficients of the national wage ranking of occupations with the overall wage ranking, and number of occupations with valid observations

	**Standardized hourly wages (USD)**				**Ratios and correlations**		
	**Mean wage**	**SD mean wage**	**Min. wage**	**Max. wage**	**Ratio max/min wage**	**Correlation with overall wage rank**	**Occupations with valid observations ( *n *)**
Netherlands	25.91	12.27	17.65	64.90	3.68	0.88	16
United Kingdom	25.53	10.62	12.93	54.95	4.25	0.84	16
United States	25.06	16.71	10.54	71.01	6.74	0.87	14
South Africa	23.58	11.73	5.52	49.59	8.98	0.87	15
Belgium	22.03	14.79	12.17	72.85	5.98	0.91	16
Germany	19.53	5.25	12.14	29.85	2.46	0.69	15
Sweden	18.86	4.13	15.26	29.97	1.96	0.68	11
Spain	16.19	5.98	8.01	32.59	4.07	0.81	15
Finland	14.59	4.66	10.77	28.66	2.66	0.86	14
Chile	13.50	7.53	5.53	33.23	6.01	0.88	15
Colombia	11.71	5.50	6.07	26.49	4.37	0.54	15
Mexico	11.49	4.81	4.70	21.51	4.57	0.65	16
Czech Republic	10.64	2.42	7.03	14.41	2.05	0.81	15
Argentina	9.89	3.26	4.60	16.07	3.50	0.85	16
India	7.69	4.87	2.45	17.68	7.22	0.50	14
Belarus	7.54	2.86	2.73	13.11	4.80	0.75	16
Brazil	6.20	4.03	2.00	19.31	9.66	0.91	16
Poland	4.93	1.34	2.77	7.68	2.77	0.67	14
Russian Fed.	4.12	1.72	1.90	7.17	3.79	0.73	15
Ukraine	3.25	0.90	1.83	4.67	2.55	0.48	16
Total	14.06	10.55	1.83	72.85	39.81		300

Source: Data from the *WageIndicator* survey 2008–2011 (weighted within countries), aggregated for 16 HRH occupations in 20 countries (*n*=300 occupation/country cells, which are not weighted across countries for the size of the national population or the national HRH workforce).

For the wage ranking of the 16 occupational groups in the HRH workforce in each country, the ranking system is from lowest to highest value. The median standardized USD gross hourly wages within each occupation/country cell were ranked from 1, indicating the occupation with the lowest median wage in the country, to 16, indicating the occupation with the highest median wage in the country (second panel of Table 1). For reasons of comparability, the ranking in countries with observations for fewer than 16 occupations was scaled between 1 and 16. Not surprisingly, the occupational group Medical Doctors ranks highest in seven of the 20 countries and ranks highest in the 20-country wage ranking (Table 1, column 6). It has the second-highest rank in another seven countries (see Additional file 3). The occupational groups for Dentists and Pharmacists rank second and third highest. In contrast, the Personal Care Workers group is ranked lowest across the 20 countries. This group has the lowest wage ranking in 13 of the 20 countries, and is ranked second-lowest in another three countries. The 20-country wage rank of the Health Care Administration & Operations occupations (non-managerial) is the second-lowest. In most countries, the Health Care Managers group has the second-highest ranking (Table 1, column 6). In almost all countries, the Health Care Managers group has lower median earnings than the Medical Doctors group, but in six countries, they have higher earnings, namely in Belarus, Czech Republic, India, the Russian Federation, and Ukraine (see Additional file 3). Arguably, ideologies of centralized state-governance historically played a more dominant role in some of these countries than did quality of care [27]. At 8.0, the Nursing & Midwifery Professionals group is in the middle of the earnings ranking (Table 1, column 6). In six countries, this occupation is ranked near the bottom at places 3 or 4, namely in Belarus, India, Mexico, Poland, the Russian Federation, and Ukraine. In contrast, this group has relatively high rankings in Brazil, Chile, the Netherlands, and Spain (see Additional file 3).

To investigate the similarity of the wage rankings, the 16-occupation ranking in each country was correlated to the overall 20-country ranking, thereby indicating how much the country’s ranking fits into the overall ranking. The second-last column in Table 2 shows the results. It depicts that the correlations are fairly high for most countries. In 11 countries (Argentina, Belgium, Brazil, Chile, Czech Republic, Finland, the Netherlands, RSA, Spain, UK, and USA) the correlations are between 0.81 and 0.91. In six countries, correlations are between 0.65 and 0.75 (Belarus, Germany, Mexico, Poland, the Russian Federation, and Sweden), and in three countries correlations are between 0.48 and 0.54 (Colombia, India, Ukraine). In conclusion, in the vast majority of countries in this study, the ranking is fairly similar. The latter countries are not a group of higher income countries or countries from one continent. Explanations for the relatively low correlations of the three countries might be linked to their qualifications and/or professional credentialing systems, but other explanatory factors may also play a role. For India, the factor of a large under-representation of people aged >40 years may be part of the explanation. Further investigations are needed to understand the differences between the countries.

For the within-country and within-occupation wage dispersion, we used two measures notably the ratio between the highest and lowest wages and the standard deviation of the median wages. Table 1 (column 3) shows the wage dispersion for the HRH occupations, and Table 2 (column 3) does so for the countries. For the HRH occupations, the standard deviation is largest for Dentists and smallest for Personal Care Workers. Comparing the countries, the standard deviation is largest in USA and smallest in Ukraine. Within countries, the ratio of the highest paying occupation to the lowest paying occupation (Table 2, column 6) shows that the wage gap is largest in Brazil, where the median wage of the highest paid HRH occupation is 9.7 times the median of the lowest paid HRH occupation, followed by South Africa. In contrast, Sweden, the Czech Republic, and Germany show more egalitarian dispersion as far as the median wages in the HRH workforce are concerned (ratios between 2.0 and 2.5).

In some countries, bonuses contribute for a substantial part to the income. The data allowed us to explore the incidence of bonuses. As explained in the previous section, we distinguished four categories of bonuses, namely fixed and variable annual bonuses as well as fixed and variable regular bonuses. Table 3 reveals the 20-country average of the proportion of these bonuses for the 16 HRH occupations. It shows that the fixed annual bonuses are the most frequent, with an average percentage between 17% and 39%. These bonuses are reported in almost all countries, with the exception of Personal Care Workers, for whom no bonus is reported in seven of the 20 countries. The second most frequently reported bonus is the variable regular bonus with an average percentage between 3% and 11%. In 11 of the 20 countries, Physiotherapists did not report receiving this bonus. Finally, the percentages of variable annual and fixed regular bonuses are the least frequently reported. In all occupations, the average is <10%. In all occupations also, the percentage of fixed bonuses as a percentage of the total bonuses is at least 67% (last column in Table 3). Pharmacists have the lowest percentage fixed bonuses in total bonuses, whereas Community Health Workers have the highest. When comparing countries (see Additional file 3), fixed annual bonuses are most common in the Netherlands, Germany, Belgium, Finland, but also in Mexico, whereas they are least common in Poland and Spain. Fixed regular bonuses are most common in Belarus. Variable annual bonuses are most often reported in Mexico, and variable regular bonuses are most common in the Czech Republic.

**Table 3:** 20-country mean of the national proportions of job holders in 16 HRH occupations reporting to receive a bonus in four bonus categories, number of countries with 0% of the job holders reporting the bonus, and proportion of fixed bonus in total bonus

	**Fixed annual bonuses**	**Countries with 0% ( *n *)**	**Fixed regular bonuses**	**Countries with 0% ( *n *)**	**Variable annual bonuses**	**Countries with 0% ( *n *)**	**Variable regular bonuses**	**Countries with 0% ( *n *)**	**Fixed bonus in total bonus**
Medical Doctors	0.23	0	0.06	5	0.03	7	0.08	3	0.73
Nursing & Midwifery Professionals	0.36	0	0.09	6	0.03	5	0.10	3	0.79
Dentists	0.17	3	0.07	7	0.02	8	0.03	8	0.82
Pharmacists	0.35	2	0.03	13	0.07	6	0.11	8	0.67
Envir. & Occ. Health Professionals	0.39	0	0.09	7	0.03	10	0.06	8	0.83
Physiotherapists	0.26	0	0.07	11	0.01	13	0.06	11	0.82
Other Health Professionals	0.29	0	0.05	7	0.02	9	0.06	3	0.82
Medical and Pharmac. Technicians	0.36	1	0.04	6	0.04	5	0.11	1	0.73
Nurses & Midwifery Associate Prof.	0.30	0	0.08	6	0.01	10	0.09	1	0.79
Community Health Workers	0.30	2	0.06	9	0.00	18	0.05	8	0.87
Other Health Associate Profess.	0.30	0	0.07	6	0.02	8	0.09	0	0.77
Personal Care Workers	0.22	7	0.03	13	0.02	14	0.05	7	0.77
Health Researchers & Educators	0.32	1	0.05	7	0.04	5	0.09	4	0.74
Health Care Managers	0.33	0	0.08	7	0.04	4	0.10	3	0.74
Health Care Admin. & Operations	0.29	1	0.05	6	0.03	4	0.08	0	0.77
Health Informatics Technicians	0.33	0	0.03	6	0.05	1	0.10	1	0.71
Total	0.30		0.06		0.03		0.08		0.77

Source: Data from the *WageIndicator* survey 2008–2011 (weighted within countries), aggregated for 16 HRH occupations in 20 countries (*n*=300 occupation/country cells, which are not weighted across countries for the size of the national population or the national HRH workforce).

### Explaining wage ranking and wage levels

Research objective 2 aimed to investigate the extent to which gender, age, and employment status composition as well as the bonuses within the occupation/country cells explain the within-country wage rankings and wage levels in standardized USD. Before turning to the results of the analysis, we briefly describe the characteristics of the 300 occupation/country cells (Table 4). The gender composition of the health sector occupations varies largely across the 20 countries. With a 20-country mean of 23% of women, the occupation of Health Informatics Technician is the most male-dominated. This is the case in 14 of the 20 countries. Medical Doctors and Environmental and Occupational Health Professionals rank second as most male-dominated occupations (both 32% women). In contrast, five occupational groups, namely Nursing & Midwifery Professionals, Physiotherapists, Nurses & Midwifery Associate Professionals, Community Health Workers, and Personal Care Workers in Health Services compete for being most strongly female-dominated. Across countries (not shown in the table), the 16 HRH occupations are most feminized in Finland and Sweden with an average of 73% women across the occupations, followed by Ukraine (69%), whereas these occupations are least feminized in India (37%) and in Mexico (42%).

**Table 4:** **20-country mean of the national proportions of job holders in 16 HRH occupations aged 30 or less, aged 50 and over, women, and employees (*versus *** **self-employed)**

	**Proportion job holders aged 30 years or less**	**Proportion job holders aged 50 years and over**	**Proportion women**	**Proportion employees**
Medical Doctors	0.13	0.27	0.32	0.81
Nursing & Midwifery Professionals	0.19	0.19	0.85	0.99
Dentists	0.20	0.22	0.46	0.61
Pharmacists	0.20	0.16	0.67	0.96
Envir. and Occup. Health Professionals	0.17	0.21	0.32	0.96
Physiotherapists	0.35	0.17	0.74	0.90
Other Health Professionals	0.25	0.20	0.74	0.93
Medical and Pharmaceutical Technicians	0.25	0.16	0.65	0.97
Nurses & Midwifery Associate Professionals	0.17	0.21	0.87	0.98
Community Health Workers	0.23	0.20	0.78	0.99
Other Health Associate Professionals	0.23	0.19	0.75	0.97
Personal Care Workers in Health Services	0.19	0.21	0.82	0.96
Health Researchers & Educators	0.26	0.18	0.59	0.96
Health Care Managers	0.11	0.22	0.64	0.97
Health Care Administration & Operations	0.19	0.20	0.72	0.98
Health Informatics Technicians	0.37	0.10	0.23	0.97
Total	0.22	0.19	0.64	0.94

Source: Data from the *WageIndicator* survey 2008–2011 (weighted within countries), aggregated for 16 HRH occupations in 20 countries (*n*=300 occupation/country cells, which are not weighted for size of country or size of HRH workforce).

The proportion of workers aged 30 years and younger in the HRH occupations varies greatly across the 20 countries (Table 4). On average, the Health Informatics Technician occupation has the largest proportion of job holders aged 30 years and younger. Across the 16 occupations, South Africa has on average the highest proportion of job holders aged <30 years (33%), followed by Brazil, whereas the Czech Republic and the Russian Federation have the lowest proportion aged <30 years (all at 15%) (see Additional file 3). The proportion of workers aged 50 years and older in the HRH occupations also varies across the 20 countries. On average, Medical Doctors have the largest proportion of job holders aged 50 years and over. Turning to the employment status of employee *vs*. self-employed, the proportion of employees is lowest for Dentists, with a 20-country mean of 61%, followed by Medical Doctors (81%). Employee status is highest for Nursing & Midwifery Professionals and Community Health Workers (99%). Across the 16 HRH occupations, the percentage of self-employed is highest in Argentina and lowest in Belarus (see Additional file 3).

To investigate the extent to which gender, age, and employment status composition as well as the bonuses affect the wage structure, we conducted OLS regressions with the wage ranks and the wage levels of the HRH occupations within the countries as the dependent variables (Table 5). The results show that occupations with a large share of job holders aged 30 years or younger have a lower wage rank, whereas no significant relations are found for cells with a large share of job holders aged 50 years and over on wage rank. Not surprisingly, the results reveal a large relation to the share of women in the occupational group: one additional percentage point of women is associated with a decrease in the wage rank of 8% on the scale from 16 to 1. Finally, the percentage of employees in an occupation is associated with a decrease in its wage rank. No significant relations were found with the wage ranks for any of the bonuses. The results concerning the wage levels reveal a slightly different picture (now the bonuses are not included, because the hourly wages explicitly include the regular bonuses and exclude the variable bonuses). The age composition does not show a significant relation to the wage levels, but the feminization of the occupations is associated with decreases in wage levels. Moreover, employment status shows a stronger relation: the percentage of employees in an occupation is associated with substantial decreases in wage levels. These findings are fairly robust. Removing, for example, the country with the largest wage dispersion from the analysis of the HRH wage levels, the coefficients, and their significance levels hardly change.

**Table 5:** **Effect of an occupation’s national proportion of job holders aged 30 years or under, job holders aged 50 years and over, women, employees (*versus *** **self-employed), and four types of bonuses on its wage rank and its wage level within countries (unstandardized coefficients and standard errors of OLS regressions)**

	**Wage rank (1=lowest, 16=highest)**			**Wage level (USD)**		
	**B**	**Sign**	**s.e**	**B**	**Sign**	**s.e**
(Constant)	23.12	***	2.27	53.95	***	5.43
Proportion aged 30 years or less	−7.53	***	2.07	−9.27		4.83
Proportion aged 50 years and over	0.88		2.23	3.55		5.32
Proportion women	−7.80	***	1.05	−8.02	**	2.46
Proportion employees	−9.44	***	2.33	−35.54	***	5.35
Proportion receiving fixed annual bonus	0.97		1.26			
Proportion receiving fixed regular bonus	0.46		2.73			
Proportion receiving variable annual bonus	7.60		4.83			
Proportion receiving variable regular bonus	2.40		2.40			
Adj R Sq	.29			.21		
N	300			300		

Source: Data from the *WageIndicator* survey 2008–2011 (weighted within countries), aggregated for 16 HRH occupations in 20 countries (*n*=300 occupation/country cells, which are not weighted across countries for the size of the national population or the national HRH workforce).

Significance levels: ***P <0.001; **P<0.005; *P<0.010.

## Conclusion and discussion

This paper breaks new ground by investigating for the first time the wage levels and the wage distribution of 16 occupational groups in the Human Resources for Health (HRH) workforce for 20 countries. Cross-country worldwide wage comparisons have not been undertaken in such great detail for occupational breakdowns before, whereas these data are needed for setting national policy in regard to healthcare provision and workforce composition as well as for international mobility in the HRH workforce. For the analyses, the data of the worldwide, continuous *WageIndicator* web survey on work and wages for 2008, 2009, 2010, and 2011 was pooled, selecting 16 occupational groups in the HRH workforce across 20 countries. The wages were controlled for purchasing power parity (PPP) in the respective years, and afterwards set to the level of 2011. In total, the micro-data included 49,687 observations. These were aggregated into 300 occupation/country cells with information about the median wages and the proportions of women, age groups, employees, and reported bonuses.

Research question 1 assumed that the ranking of the median wages in the 16 occupational groups was similar across the 20 countries. Medical Doctors were found to have the highest 20-country wage rank and Personal Care Workers group the lowest. Medical Doctors rank highest in seven of the 20 countries and second-highest in a further seven countries. Personal Care Workers rank lowest in 14 of the 20 countries and second-lowest in a further three countries. In six countries, five of which are post-Soviet countries, the Health Care Managers group has higher median earnings than the Medical Doctors group. The Nursing & Midwifery Professionals group is in the middle of the earnings ranking (rank 10). In six countries, these occupations are ranked near the bottom, whereas this group has relatively high rankings in four other countries.

Research objective 1 also aimed to investigate the extent to which countries are similar with respect to the wage levels and wage dispersion, and the incidence of bonuses in the HRH occupations. Large wage differences were revealed, for example: a Ukrainian doctor earns 20 times less than his American counterpart. A similar pattern can be observed for Ukrainian Nursing & Midwifery Professionals, who earn 11 times less than their Dutch counterparts, and Ukrainian Personal Care Workers earn nine times less than Personal Care Workers in the Netherlands. The standard deviation across the 16 occupations is largest in the USA, and the ratio of highest to lowest earning occupation is largest in Brazil, whereas Sweden shows the most egalitarian wage dispersion in its HRH workforce. The fixed annual bonuses are most common across countries and across occupations, compared to variable or regular bonuses. Practices concerning variable bonuses are in all country/occupation cells reported by <10% of respondents.

Research objective 2 aimed to investigate the extent to which gender, age, and employment status composition as well as the bonuses within the occupation/country cells explain their within-country wage rankings and wage levels in standardized USD. The gender composition of the health sector occupations varies largely across the 20 countries, with Health Informatics Technicians, Medical Doctors, and Environmental and Occupational Health Professionals as the most male-dominated occupations, and the Nursing & Midwifery Professionals, Physiotherapists, Nurses & Midwifery Associate Professionals, Community Health Workers, and Personal Care Workers in Health Services the most female-dominated occupations. The 16 HRH occupations are most feminized in Finland, and least feminized in India. Across countries, the percentage of self-employed is highest in the Dentist occupation and lowest in Nursing & Midwifery Professionals and the Community Health Workers. The percentages of women and self-employed in the occupation/country cells are highly significantly related to their wage ranks and wage levels. One additional percentage point of women is associated with a decrease in the wage rank of 8% on the scale from 16 to 1 and 8% in standardized USD. This result is in line with the devaluation theory, arguing that ‘female’ tasks and skills are devaluated on the labor market which is mainly mirrored in the fact that with an increase of women in occupation wages become lower [28]. While previous studies mainly focused on the US or Europe, this study is one of the first showing this devaluation effect at a global level. Age composition and bonuses are hardly related to the wage structure.

Finally, certain limitations of the study must be mentioned. The first one relates to the data stemming from a volunteer web survey. As indicated, a comparison with labor force data has revealed that individuals aged 40 years and over are most under-represented across all countries, but particularly for India. This holds noticeably more for women than for men. To minimize the sample bias, within-country weights have been applied. Further, considering the lack of randomly sampled data in this area, it seems worthwhile to emphasize the argument made by Couper and Miller [29] according to which it is better not to treat survey quality as an absolute, but to evaluate quality relative to other features, such as the availability of better data, the research design, and the stated goals of the survey. The second limitation relates to the definition of wages. *WageIndicator* applies a standard definition to all countries and occupations, as explained in the Methods section. However, wage structures may vary across countries and may include non-financial remunerations, such as housing, food, transportation cost reimbursements, employer-paid health insurance, social premiums, or pension contributions. Thus, there may be additional sources of variation across countries which are not taken into account, but certainly call for further research. A third limitation relates to the occupational titles in this study, which are assumed to refer to the same job content across countries. Thus, the occupational group of Nursing & Midwifery Professionals is assumed to have the same set of tasks across the world. However, so far the job content of the HRH occupational groups has not been empirically tested on a worldwide scale and would require a separate project to develop such testing. A fourth limitation relates to the diploma credentials in the HRH occupations. In most countries, credentials are required for most HRH occupations. Depending on the supply and demand ratio in the local labor market, these credentials will or will not be required for entry into a job. In most workplaces, credentials will lead to higher earnings. However, the current dataset does not allow controlling for credentials and therefore the wages of accredited *versus* non-accredited job holders in the same occupational group could not be studied.

Despite these limitations, this explorative study contributes to the understanding of wage levels and wage dispersion in the HRH field. It is the first study on wages in a wide range of HRH occupations and a wide range of countries in four continents. Although it can be criticized that a random sample of the health sector workforce across the 20 countries would have generated more representative findings, it should be taken into account that such surveys are extremely expensive and mostly one-time data collections. In this context, the substantially lower costs, the continuity, and the actuality of the *WageIndicator* data collection shows the potential of this data to yield valuable insights into a complex and diverse landscape of wage rankings, dispersions, and standardized wages. Moreover, the survey data allow for extending the analyses of wage structures to other components of healthcare labor markets, such as the impact of wage levels on labor market behavior, as has been undertaken for nurses in four countries [30], or for the drivers of migration in the health workforce.

## Abbreviations

HRH: Human Resources for Health; ILO: International Labour Organization; ISCO: International Standard Classification of Occupations; OWW: October Inquiry and the Occupational Wages; PPP: Purchasing Power Parity; USD: US Dollar.

## Competing interests

The authors declare no competing interests.

## Authors’ contributions

KT contributed to the analyses of the survey data, DHdV contributed to the defining of the health sector occupations, and SS contributed to the analyses of the selection bias in the data set. All authors reviewed and approved the final manuscript.

## Authors’ information

KT is a Research Coordinator at Amsterdam Institute of Advanced Labor Studies (AIAS) at the University of Amsterdam, and a Professor of Women and Work at the Department of Sociology, Erasmus University Rotterdam. She is the scientific coordinator of the continuous *WageIndicator* web survey on work and wages. Her research interests are wage setting processes, working time, and occupations.

DHdV is a Researcher at the Department of Sociology and Anthropology at the University of Amsterdam, and affiliated to the Center for Social Science and Global Health and AIAS. He previously was Research and Evaluation Manager at USAID’s Capacity Project, a human resources for health strengthening project.

SS is an Assistant Professor at the Department of Sociology and Anthropology at the University of Amsterdam and an affiliated senior researcher at the Erasmus Studio Rotterdam. Her main research interests are (web) survey methodology, gender inequalities, comparative labor market research, and quantitative research methods.

## Supplementary Material

Additional file 1 **Stylized *WageIndicator *** **questionnaire (pdf).** Click here for file

Additional file 2 **Mapping the selected *WageIndicator *** **occupations into the 16 HRH occupations and their ISCO-08 codes (MS Excel).** Click here for file

Additional file 3 The aggregate data file of the 16 occupations * 20 country cells (SPSS). Click here for file
