# Supplementary material for: Health workforce remuneration: comparing wage levels, ranking, and dispersion of 16 occupational groups in 20 countries
Source: Hum Resour Health. 2013 Feb 28;11:11. doi: 10.1186/1478-4491-11-11 (PMC3787851; doi:10.1186/1478-4491-11-11)
Supplement: Additional file 1 — Stylized WageIndicator questionnaire (pdf). [file 1478-4491-11-11-S1.pdf]

The stylized *WageIndicator* questionnaire

a worldwide, continuous, multilingual web-survey on work and wages with paper supplements

www.wageindicator.org

Kea Tijdens, University of Amsterdam / AIAS (Netherlands)

Sanne van Zijl, LadyQ data management, Werkhoven (Netherlands)

August 19, 2010

This paper presents the stylized *WageIndicator* questionnaire. The word 'stylized' implies the following:

- The stylized questionnaire includes the most important questions, and does not show all questions and all features, because this paper would otherwise count hundreds of pages
- The stylized questionnaire shows the routings for employees only and not for other employment status groups
- The stylized questionnaire does not show the conditional skips; where needed for readability, the text of the survey question has been adapted.
- The stylized questionnaire does not include project-related questions
- The stylized questionnaire does not include country-specific questions
- The stylized questionnaire refers with the word '_list' in the **RESPONSE/VALUE** column to the several search trees, e.g. for education, region, and the like
- The stylized questionnaire does not indicate which questions are compulsory
- The stylized questionnaire does not show which questions are switched on/off per country
- The stylized questionnaire does not show how the survey is divided across web-pages
- The stylized questionnaire does not indicate which grid questions include random items

The colours indicate the following:

- Dark green with white letters indicates the headings of the survey questions
- Dark green indicates the survey questions
- Light green indicates the answers
- Grey with white letters indicates the variable names
- Pink indicates the hints or alerts

****

The stylized *WageIndicator* questionnaire

VARIABLE NAME	SOURCE LABEL	RESPONSE/VALUE
	Why complete the questionnaire?	
contst71	What is your employment status?	
HINT	If you have more than one job, please answer the questionnaire with reference to your MAIN job	
	Employee	140
	Civil servant	141
	Employee on permanent contract	146
	Employee on fixed-term contract	147
	Self-employed, own-account worker, freelance	202
	Self-employed with personnel	201
	Paid family worker / working for family business	103
	Working without pay (family business or farm)	306
	Unpaid voluntary worker	307
	Housework	308
	Looking for a job / unemployed	410
	Sickness benefit / incapacity for work	515
	Retired	116
	School pupil	620
	Student in full-time education	720
	Apprentice	825
	In a job creation scheme	130
	In military or comparable services	131
	Retired	916
	Never had a job	1
	Other	950
sex	Are you a woman or a man?	
	Woman	1
	Man	2
countryselect	In which country do you live?	COUNTRY_list
regiwocheck	Do you work outside [COUNTRY OF SURVEY]?	y_n
regihom1	Where do you live?	REGION_list
nrofjobs	Do you have one paid job or more?	
	One job	1
	Two jobs	3
	Three or more jobs	4
HINT	Please complete the questionnaire for your MAIN job	
controth	Is your workplace based in another company than the one that is paying your salary?	
	No	0
	No, I am on secondment	5
	Yes, I am a posted worker	2
	Yes, I am a temporary agency worker	1
	Yes, I am working for a labour contractor	6
	Yes, other	3
HINT	Please complete the questionnaire for your current workplace	
occupai	What is your occupation?	OCCUPATION_list
HINT	If your occupation is not in the list, please select the one that comes closest	
occtype	Do you want to specify your occupation in greater detail?	
	No, the job title is alright	1
	Assistant	2
	Junior	3
	Apprentice	4
	Trainee	5
	Trainer, training responsibilities	6

VARIABLE NAME	SOURCE LABEL	RESPONSE/VALUE
	Foreman, forewoman	7
	Senior	8
	Team leader	9
	Supervisor	10
	Head	11
	Other	99
occtext	If you want to specify your occupation more accurately, please do so here	text
depmale	Are co-workers in similar positions mostly male?	y_n
supv0	Do you have a supervisory position?	y_n
HINT	This applies to people working directly under your supervision	
SUPV	How many people work directly under your supervision?	0-10000
SUPVWOM	How many of these people are women?	0-10000
sector	What is your main business activity?	INDUSTRY_list
firmsize	How many people are employed at your place of work?	
HINT	Applicable to your establishment	
	0 (self-employed without personnel)	0
	1 - 4	11
	5 - 9	12
	10 - 20	2
	20 - 50	3
	50 - 100	4
	100 - 200	5
	200 - 500	6
	500 - 1.000	7
	1.000 - 2.000	8
	2.000 - 5.000	9
	5.000 or more	10
firmfema	What percentage of employees are female?	
HINT	Applicable to your establishment	
	0 - 20 %	1
	20 - 40 %	2
	40 - 60 %	3
	60 - 80 %	4
	80 - 100 %	5
	I don't know	-7
	How many employees in your organisation are	
HINT	Applicable to your establishment	
ILC013a	Under age 25	
	0 - 20 %	1
	20 - 40 %	2
	40 - 60 %	3
	60 - 80 %	4
	80 - 100 %	5
	I don't know	-7
ILC013b	Between 25 and 50	
	0 - 20 %	1
	20 - 40 %	2
	40 - 60 %	3
	60 - 80 %	4
	80 - 100 %	5
	I don't know	-7
ILC013c	Over 50	
	0 - 20 %	1
	20 - 40 %	2
	40 - 60 %	3
	60 - 80 %	4
	80 - 100 %	5
	I don't know	-7

VARIABLE NAME	SOURCE LABEL	RESPONSE/VALUE
firmloca	Is your organisation established in more than one location?	
	No	0
	Yes, in 1 city / municipality	1
	Yes, in 1 region	2
	Yes, in 2 or more regions	3
	Yes, throughout the country	4
	Yes, throughout the country and abroad	5
	Yes, but only abroad	6
firmowne	Is your organisation domestic- or foreign owned?	
	Wholly domestic owned	1
	Partly domestic owned, partly foreign owned	2
	Wholly foreign owned	3
	I don't know	-7
	Not applicable	-8
firmow01	Please select the country where the foreign investment comes from	COUNTRY_list
firmpriv	Do you work ...	
	In the private sector	1
	In the public sector	2
	In the not-for-profit sector	3
	In a co-operative organisation	7
	In a family business	8
	I don't know	-7
	Other	99
caocate3	Is your occupational category ...	
	White collar worker (clerical, administrative)	2
	White collar worker (with exception of civil servants)	8
	Blue collar worker	1
	Civil servant	5
	Apprentice, trainee	4
	Professional, managerial or technical staff	3
	Contractual	6
	Other	99
caofirm1	Are you covered by a collective agreement about wages?	
	Yes	1
	Yes, but I myself am not covered	5
	No	0
	I don't know	-7
caofir91	If yes, are your wages predominantly determined by this agreement?	y_n_dk
caofirm8	If yes, please, can you specify the collective agreement?	
	Agreement at company level	2
	Sectoral level agreement	1
	Governement directive / wage board	3
	Municipal or intermunicipal level agreement	20
	Regional level agreement	22
	National level agreement	23
	Combination of contracts	24
	I don't know	-7
caofirm9	Are fringe benefits negotiated as well in this agreement?	y_n_dk
educat	What is your highest level of education?	EDUCATION_list
HINT	If you are currently studying, please indicate the highest level of education you reached. If you went to school abroad enter the equivalent level.	
yybirth	In which year were you born?	1930-YEAR_NOW-10
yyeduca	In which year did you finish continuous full-time education?	1950-YEAR_NOW+10
HINT	PhD graduates, please complete the year you obtained your PhD	
edujobtr	How much time is needed to become fully effective in your job for someone with your qualifications?	
	No training period required	0
	1 - 2 days	1

VARIABLE NAME	SOURCE LABEL	RESPONSE/VALUE
	3 - 6 days	2
	1 - 4 weeks	3
	1 - 3 months	4
	3 - 6 months	5
	0.5 - 1 year	6
	1 year or more	7
	I don't know	-7
training	Over the past 12 months, how much training did you get paid for or provided by your EMPLOYER, in order to improve your skills?	
	None	0
	1 - 2 days	1
	3 - 6 days	2
	1 - 2 weeks	3
	3 - 4 weeks	4
	1 - 2 months	5
	2 months or more	6
	Not applicable	-8
trainin1	Over the past 12 months, how much training have you received, paid for YOURSELF in order to improve your skills?	
	None	0
	1 - 2 days	1
	3 - 6 days	2
	1 - 2 weeks	3
	3 - 4 weeks	4
	1 - 2 months	5
	2 months or more	6
	Not applicable	-8
educapp0	Have you ever been on an apprenticeship?	
	No	0
	Yes, during my full-time education	1
	Yes, following my full-time education	2
	Yes, but not immediately following my full-time education	3
educlevl	Do your qualifications match your job?	
	Yes	1
	No, I am overqualified for my job	3
	No, I am underqualified for my job	2
	I don't know	-7
eduexpe	If you are underqualified, does your experience compensate for that?	
	No	0
	Yes, to a minor degree	1
	Yes, more or less	2
	Yes, almost fully	3
	Yes, completely	4
	I don't know	-7
contrac5	Do you have a permanent contract?	y_n
contract	If not, what kind of employment contract do you have?	
	Fixed-term contract with the prospect of permanent employment	2
	Fixed-term contract without the prospect of permanent employment	3
	Apprentice	6
	On-call contract	4
	Community work scheme / subsidised employment	14
	In community or military service	15
	Contract with temp agency	7
	Seasonal work	9
	Fixed-term contract to cover another worker	21
	Year contract	30
	Contractor for labour with equipment or vehicle	31
	Combination of contracts	11
	Other type of contract	99

VARIABLE NAME	SOURCE LABEL	RESPONSE/VALUE
hrshisto	Do you work full-time?	y_n
hrsdayp2	How many days a week do you usually work?	1-7
hrscont3	Are your working hours agreed with your employer?	
	Yes, in writing	1
	Yes, verbally	2
	No	0
	Not applicable	-8
hrscontr	How many hours a week do you work under the terms of your contract?	0.15-100.45
hrsreal0	Do you usually work the number of hours laid down in your contract?	
	Yes	1
	No, I work less hours	2
	No, I work more hours	3
	Not applicable	-8
hrsreal	How many hours a week do you usually work?	0.15-100.45
	About your working hours	
tiroster	I work shifts or irregular hours	y_n
tisatday	I regularly work on Saturdays	y_n
tisunday	I regularly work on Sundays	y_n
tievning	I regularly work in the evenings	y_n
timornin	In the past month, I have began with my working day before six o'clock in the morning	y_n
tinight	In the past month, I have ended with my working day after midnight	y_n
wageper0	Do you receive your salary per [MONTH]?	y_n
wageboth	Do you know your GROSS and your NET wage?	
	Yes, my gross and my net wage	1
	Only my gross wage	2
	Only my net wage	3
	What was your last wage?	
HINT	This applies to your LAST wage including allowances, variable income elements, holiday allowance, expense allowance or overtime	
wagegr1	Gross wage [VALUTA]	amount
wagene1	Net wage [VALUTA]	amount
wagetext	If you want to specify your payment period, please do so here	text
wageregu	Did you receive your last wage	
	[Bank account / By cheque / Bank draft]	1
	Cash in hand	2
	In kind	4
	Combination	5
wagetax1	Do you contribute to social security?	y_n
wagetax2	Is your contribution to social security calculated on the basis of your whole wage or only on a part of it?	
	On the whole	1
	On a part	2
	I don't know	-7
	Not applicable	-8
	More about your wage	
wageonti	You received your last wage on time	y_n_na
wageincr	I have received a pay raise in the past 12 months	y_n_na
wagetop	I have reached the top of my grade	y_n_na
wageask	I have asked for a pay raise in the past 12 months	y_n_na
paydiscu	I have had a frank discussion about my pay with my employer or supervisor in the past 12 months	y_n_na
wagevary	My wage varies from month to month	y_n_na
seekwage	I could earn more with another employer	y_n_na
wageperf	I would prefer if my wage depended more on my performance	y_n_na
seekeasy	I could easily find another job	y_n_na
caoimpo1	I think it is important that my wages are covered by a collective agreement	y_n_na
	Did your last wage include any of the following allowances? If so, how much?	

VARIABLE NAME	SOURCE LABEL	RESPONSE/VALUE
washifts	Shift / unsocial hours / weekend allowance	y_n_na_amount
waovrtm	Overtime bonus / premium	y_n_na_amount
wadirty	Dirty or dangerous work allowance / Inconvenience or hardship allowance	y_n_na_amount
watips	Tips	y_n_na_amount
wasenior	Seniority / managerial bonus	y_n_na_amount
watarget	Target related allowance / Personal performance allowance	y_n_na_amount
waperfom	Commission	y_n_na_amount
waannual	Annual bonus	y_n_na_amount
wayearnd	End-of-year bonus, Christmas bonus	y_n_na_amount
wahollid	Holiday allowance	y_n_na_amount
wagroupb	Group performance allowance / Team or departmental bonus	y_n_na_amount
wacommut	Commuting / public transport allowance	y_n_na_amount
waprofit	Profit sharing	y_n_na_amount
wacityal	City compensatory allowance	y_n_na_amount
waother	Other allowances	y_n_na_amount
	In the past 12 months, have you been a participant in the following work-related schemes or benefits?	
frexpe61	Arrangements regarding expences	y_n_na
frcar61	Transport arrangements (lease car, company car, commuting costs)	y_n_na
frlapt61	PC at home / laptop	y_n_na
frdayc61	Children's day-care arrangements	y_n_na
frmedi61	Health insurance arrangements	y_n_na
frpens61	Pension schemes	y_n_na
frsavs61	Savings schemes	y_n_na
frunem61	Unemployment schemes	y_n_na
frshar61	Share options arrangements	y_n_na
frhous61	Contribution to lodging / costs related housing	y_n_na
frmort61	Lower rate of interest on mortgage	y_n_na
frspor61	Sports facilities	y_n_na
pacante	Canteen/food voucher	y_n_na
frgdik61	Benefits in kind (vouchers and discounted goods and services)	y_n_na
frothe61	Other additions to pay from your employer over the past 12 months	y_n_na
	Do you receive any of the following annual bonuses?	
frnhol61	Holiday allowance	y_n_na
frnend61	End-of-year bonus	y_n_na
frnpro61	Profit share	y_n_na
frncoo61	Payments from balance surplus in co-operatives	y_n_na
frnfes61	Festival bonus	y_n_na
frnper61	Performance bonus	y_n_na
frnoth61	Other annual bonus	y_n_na
yyfstjob	What year did you start your first job?	1950_YEAR_NOW
jobhist	How many times have you changed your employer since?	1-100
yycuemp0	In which year did you join your current employer?	1950_YEAR_NOW
jobhist2	How many positions have you occupied with your current employer, including your current job?	1-10 or more
yycuposi	In which year did you attain your current position?	1950_YEAR_NOW
	In your organisation	
dephisto	Have you been working in different departments?	y_n_na
jobpromo	Have you been promoted?	y_n_na
	Your employment history	
breaknr	Since your first job, have you ever been without a job?	y_n
break0	If yes, how many times have you been without a job?	1-100
breakpe1	If yes, how long have you been without a job?	
	0 - 3 months	1
	3 - 6 months	2
	0.5 - 1 year	3
	1 year or more	4
break	In which year did you stop working and in which year did you return to work?	1950_YEAR_NOW

VARIABLE NAME	SOURCE LABEL	RESPONSE/VALUE
ILC019	Were you during the last 12 months absent at any time due to illness, accident or for other health reasons?	y_n_na
ILC020	If yes, how often were you absent during the last 12 months?	0-100
ILC021	If yes, how many DAYS were you absent during the last 12 months?	1-360
memtrad1	Member of a trade union?	y_n_na
memdele1	Member of a works council	y_n_na
memstaff	Member of a staff association	y_n_na
memproff	Member of a professional organisation	y_n_na
memtrd61	Of which trade union are you a member?	TRADE UNION_list
regiwor0	Is your workplace in the same region where you live?	
	Yes	1
	Yes, my workplace and my home are in the same building.	2
	No	0
	I am a mobile worker covering more regions.	3
	How long does it take to commute from your home to work?	
commtime	Time in minutes (one way)	
	0 / Not applicable	0
	0 - 15 min.	1
	15 - 30 min.	2
	30 - 45 min.	3
	45 - 60 min.	4
	1 hour - 1 hour 15	5
	1 hour 15 - 1 hour 30	6
	1 hour 30 - 2 hours	7
	2 hours or more	8
commdist	Distance in kilometers (one way)	
	0 / Not applicable	0
	0 - 5	1
	5 - 10	2
	10 - 15	3
	15 - 20	4
	20 - 25	5
	25 - 50	6
	50 - 75	7
	75 or more	8
	How do you travel to work?	
comcar	By car	checkbox
combike	By bicycle	checkbox
commoped	By moped / scooter	checkbox
combus	By bus, tram or underground	checkbox
comtrain	By train	checkbox
comfoot	On foot	checkbox
comcomt	Company provides transport	checkbox
comhome	I work from home	checkbox
comother	Other	checkbox
cobself1	Were you born in [COUNTRY OF SURVEY]?	y_n
cobself	If no, in which country were you born?	COUNTRY_list
cobmoth1	Was your mother born in the same country as you?	y_n_dk
cobmothe	If no, in which country was your mother born?	COUNTRY_list
cobfath1	Was your father born in the same country as you?	y_n_dk
cobfathe	If no, in which country was your father born?	COUNTRY_list
yyarriva	In which year did you come to live in [COUNTRY OF SURVEY] and why did you come here?	1950_YEAR_NOW
cobreaso	Came because of	
	Economic reasons	4
	Family reasons	1
	Asylum	2
	Other	99
cobethg1	To which [ethnic/social/minority] group do you belong?	ETHNIC GROUP_list

VARIABLE NAME	SOURCE LABEL	RESPONSE/VALUE
coblang1	Which language do you usually speak at home?	LANGUAGE_list
regihom2	Where do you live?	REGION_list
hhnrmemb	How many people live in your household?	1-6 or more
hhinconr	How many people in your household have an income from work, benefits or other sources?	0-6 or more
	With whom do you live in your household?	
hhpartn1	Partner	y_n
hhchild	One or more children	y_n
hhchildp	One or more children, but only for part of the week/year	y_n
hhgchild	Grandchildren	y_n
hhstate7	Parents (in-law)	y_n
hhstat10	Grandparents (in-law)	y_n
hhstat21	Brothers or sisters (in-law)	y_n
hhstat13	Joint family	y_n
hhstat20	Other relatives	y_n
hhstat12	House help	y_n
hhstat22	Employer	y_n
hhlivein	Live-in friend	y_n
hhstate8	Non-family members	y_n
hhstat7	What is your current marital status?	
	Married	1
	Widowed	2
	Divorced	3
	Never married	4
chld	Do you have children?	y_n
	If yes, how many children do you have?	
chldhome	Living at home	0-6_dk
chldout	Not living at home	0-6_dk
	In what year are your children born?	
morechld	Eldest child	1950_YEAR_NOW
yyyochld	Youngest child	1950_YEAR_NOW
ILC018	How is your health in general? Would you say it is …	
	Very good	1
	Good	2
	Fair	3
	Bad	4
	Very bad	5
	Finally, how satisfied are you with …	
satjob	Your job	satisfaction5pt_na
bgsatjob	Your work	satisfaction5pt_na
wagesat2	Your pay	satisfaction5pt_na
bgwagesa	Your income / profit	satisfaction5pt_na
commsat	Your commuting time	satisfaction5pt_na
satcombi	Your combination of work and family life	satisfaction5pt_na
satsecur	Your job security	satisfaction5pt_na
satenvir	Your work environment	satisfaction5pt_na
sathrs	Your working hours	satisfaction5pt_na
satcoll	Your relationship with your colleagues at work	satisfaction5pt_na
satsupvi	Your relationship with your superiors at work	satisfaction5pt_na
	Which of the following best describes your use of the internet at home over the past month?	
	Every day or almost every day	1
	A couple of times a week	2
	A couple of times a month	3
	Once a month or less	4
	Did not use the internet et all	5
	I don't know	-7
satlife	All things considered, how satisfied are you with your life as-a-whole now?	
	1 - Dissatisfied	1

VARIABLE NAME	SOURCE LABEL	RESPONSE/VALUE
	2	2
	3	3
	4	4
	5	5
	6	6
	7	7
	8	8
	9	9
	10 - Satisfied	10
	I don't know	-7
	Thanks for completing part 1 of the questionnaire!	
HINT	You don’t have to complete part 2 of our salary questionnaire.<br>But if you do it anyhow, we double your chance to win the prize!	
part_2	Please, take me to part 2!	y_n
	More about your occupation	
contrac4	In your second job, are you ...	
	Employee	140
	Self-employed, own-account worker, freelance	202
	Family worker / working for family business	103
	Trainee / apprentice	825
	Atypical labour contracts (temporary, interim, project, occasional)	918
	Regular wage earner	944
	Self-employed and wage earner	205
	Unpaid family worker	306
	Unpaid non-family worker	307
	Other	950
hrssecon	How many hours a week do you usually work in your second job?	0.15-100.45
	Your opinion	
jobene61	I have sufficient energy to do my job	agree5pt_na
spvsup61	I have sufficient support from my supervisor	agree5pt_na
occcho61	I am happy to work in this occupation	agree5pt_na
jobcha61	My job is sufficiently varied	agree5pt_na
jobint61	Over the past 12 months, my job has become more interesting	agree5pt_na
	About your job	
constr61	How often do you find your job stressful?	neverdaily5pt_na
conhea61	How often do you work in unhealthy conditions?	neverdaily5pt_na
condan61	How often do you work in dangerous conditions?	neverdaily5pt_na
traiww61	How often do you find training for your job would be worthwhile?	neverdaily5pt_na
conphy61	How often do you come home from work physically exhausted?	neverdaily5pt_na
conmen61	How often do you come home from work mentally exhausted?	neverdaily5pt_na
jobaut61	How often do you organise your own work tasks?	neverdaily5pt_na
conbor61	How often do you find your job boring?	neverdaily5pt_na
jobrot62	How often do you take over tasks assigned to your co-workers?	neverdaily5pt_na
jobrou62	How often do you find your job involves monotonous tasks?	neverdaily5pt_na
jobsec62	How often do you worry about your job security?	neverdaily5pt_na
	More about your education and training	
firmsiz2	How many people are employed by the whole organisation in your country?	
	Less than 10	1
	10 - 20	2
	20 - 50	3
	50 - 100	4
	100 - 200	5
	200 - 500	6
	500 - 1,000	7
	1,000 - 2,000	8
	2,000 - 5,000	9
	5,000 or more	10
	I don't know	-7
firmfem1	What percentage of employees is female?	

VARIABLE NAME	SOURCE LABEL	RESPONSE/VALUE
	0 - 20 %	1
	20 - 40 %	2
	40 - 60 %	3
	60 - 80 %	4
	80 - 100 %	5
	I don't know	-7
	In your workplace ...	
jobalone	You work alone	y_n_na
depteam	Most of your work is organised in teams	y_n_na
jobnocow	You deal directly with people who are not co-workers	y_n_na
jobfrien	You consider some of your co-workers to be friends	y_n_na
supvfema	Your direct supervisor is a woman	y_n_na
compjob	You work with a computer	y_n_na
compequi	You work with other automated equipment	y_n_na
compinet	You use the Internet	y_n_na
depdisc	It is possible to discuss changes in work with your management	y_n_na
depmach1	Machines/equipment are in a good state of repair	y_n_na
depstaf3	Staffing levels are sufficient	y_n_na
depconf3	Conflicts regularly occur	y_n_na
depwork1	Work is well organised	y_n_na
depinfo1	You are well informed about the work you have to do	y_n_na
depinfo2	You are well informed about what is going on	y_n_na
supvclo1	You are closely supervised	y_n_na
wagequa3	Women and men have equal opportunities	y_n_na
firmrela	Relations between management and employees are good	y_n_na
firmresp	You are treated with respect	y_n_na
firmtrust	You trust the management	y_n_na
firmprom	Promotions are handled fairly	y_n_na
	Your opinion	
firmgro1	Over the past 12 months, the workforce has been ...	increasing5pt_na
firmgro2	In the next 12 months, the workforce is likely to be ...	increasing5pt_na
firmhie1	Over the past 12 months, the number of managers has been ...	increasing5pt_na
firmhie2	In the next 12 months, the number of managers is likely to be ...	increasing5pt_na
	In your organization ...	
firmrep1	Is there a trade union representative?	y_n_na
firmrep2	Is there a shop steward system?	y_n_na
firmrep3	Is there a plant / unitary union structure?	y_n_na
firmcou1	Is there a works council?	y_n_na
firmcou2	Is there a works / staff council?	y_n_na
firminfl	Do the trade unions influence the way the organisation is managed?	y_n_na
firmequ1	Is there an equal opportunity committee?	y_n_na
firmrep4	Is there an officer/employee association?	y_n_na
firmrep8	Are you allowed by your organization to join a trade union/association, if you so desire?	y_n_na
jobpersp	Do you have good career opportunities?	y_n_na
jobfamil	Are any of your co-workers family members?	y_n_na
firmre10	Is there a Personnel or Human Resources department?	y_n_na
supvsex	Is your direct supervisor male or female?	
	Female	1
	Male	2
	Why are you without a job?	
break2	Illness or occupational disability / accident / disease	checkbox
break3	Unemployment / dismissed / inability to find a suitable job	checkbox
break13	I could not continue my own business	checkbox
break14	Seasonal work	checkbox
break15	Short-time arrangements	checkbox
break1	Caring for children over 1 year	checkbox
break11	Housekeeping.	checkbox
break12	Caring for other members of household	checkbox

VARIABLE NAME	SOURCE LABEL	RESPONSE/VALUE
break7	Caring for other persons	checkbox
break6	Military service or equivalent.	checkbox
break5	Other reasons	checkbox
break19	If break, did you have job protection during this period?	
	No	0
	Yes, part of the time	1
	Yes, for the whole period	2
	I don't know	-7
	If break, which income did you receive during this period?	
break14	Unemployment benefit	checkbox
break15	Leave coverage	checkbox
break16	My partner / family provided for me	checkbox
break17	Disability benefit	checkbox
break20	Only past savings	checkbox
break21	Lay off pay	checkbox
break18	Other	checkbox
breaknon	None	checkbox
seekhisb	If break, how long did it take you to find your first job after this period?	
	I returned to my previous employer	0
	I did not (have to) look for a job	1
	I started my own company / joined the family business	9
	Less than 1 month	2
	1 - 3 months	3
	3 - 6 months	4
	0.5 - 1 year	5
	1 year or more	6
	I don't know	-7
seekhist	If no break, how long did you spend looking for your first job?	
	I did not look for a job	0
	Less than 1 month	1
	1 - 3 months	2
	3 - 6 months	3
	0.5 - 1 year	4
	1 year or more	5
	I don't know	-7
occhist2	If > 1 job, are you still working in the same business activity / industry as you were in your first job?	y_n
occhist1	If > 1 job, are you still working in the same occupation as in your first job?	y_n
seekjob	Have you looked for another job in the past four weeks?	
	No, I don't want to change jobs	1
	No, but I am interested in a new job	2
	Yes, but I have not found one yet	3
	Yes, and I have found one	4
	If yes, how do you go about looking for another job?	
sea1exam	I do an official examination for a public post	checkbox
seatraun	Visit an employment center (trade unions agencies, ONG, churches and not-for-profit organisations)	checkbox
seanempl	Visit a public / national employment agency	checkbox
seafamil	Ask family and acquaintances	checkbox
seainter	Search a jobsite on the Internet	checkbox
seajnals	Look in professional magazines	checkbox
seanwprs	Via an advertisement / look in newspapers	checkbox
seapempl	Visit a private employment agency	checkbox
seaphone	Phone employers directly	checkbox
seapucon	Through public selection	checkbox
searesum	Send my CV to employers speculatively	checkbox
seascho3	Via an apprenticeship / school	checkbox
seatemp	Visit a temp agency	checkbox
seaweb	Through the Internet	checkbox

VARIABLE NAME	SOURCE LABEL	RESPONSE/VALUE
	In the past 12 months, has your organisation	
firmdis1	Announced redundancies?	y_n_na
firmpri2	Been privatised?	y_n_na
firmtrai	Offered you training opportunities?	y_n_na
firmreo1	Faced reorganisation that has affected your workplace?	y_n_na
firmprot	Faced an employee protest/strike?	y_n_na
firminit	Taken new strategic initiatives?	y_n_na
firmreti	Announced voluntary retirement?	y_n_na
firmmerg	Faced a merger with another organisation?	y_n_na
firmcom1	Renewed its computer equipment / machinery?	y_n_na
firmstra	Had a competent management strategy?	y_n_na
firmbro1	Been under immediate threat of bankruptcy?	y_n_na
	What do you expect to happen in your organisation in the next 12 months?	
firmdism	Announcement of redundancies	y_n_na
firmpri3	Privatisation	y_n_na
firmtra2	Training opportunities will be offered	y_n_na
firmreo2	Reorganisation / organisational change	y_n_na
firmmer2	Merger with another organisation	y_n_na
firmcom3	Renewal of computer equipment / machinery	y_n_na
firmstr2	Competent management strategy	y_n_na
firmbro2	Urgent threat of bankruptcy	y_n_na
firmini2	New strategic initiatives	y_n_na
firmrecr	Mass manpower recruitment	y_n_na
firmdis2	Lay off/voluntary retirement	y_n_na
jobfutu1	Do you expect to be with your current employer still in a year's time?	y_n_dk
jobfutu2	Do you expect to have a job in a year's time?	y_n_dk
jobfutu3	Do you expect to have your business still in a year's time?	y_n_dk
jobfutu4	If yes, will this be in the same position?	
	Yes, in the same position	1
	No, I will be promoted to a higher position	2
	No, I will be working in another position	3
	No, I will be demoted to a lower position	4
jobfutu5	If no, why would this not be the case?	
	I'll be working for another employer	3
	I'll be caring for children	4
	I'll be doing domestic work	13
	I'll be in (early) retirement	5
	I'll be self-employed or working for a family business	6
	Because of health problems	7
	I'll be doing further training or study	8
	I'll have been dismissed	9
	My contract will have expired	10
	My business will have gone	12
	My organisation's contract to provide the service may have expired	20
	Other reasons	11
	What do you expect to happen in the next 12 months?	
jobprom3	I am eager to get promoted	y_n_na
jobtasks	My work responsibilities are likely to change	y_n_na
jobloss	My post is likely to be made redundant	y_n_na
payrise	I will get a pay rise.	y_n_na
prefjob	I am eager to find a new job	y_n_na
comptrai	I will take computer courses to keep up with new demands at the workplace	y_n_na
preflott	I hope to win the lottery	y_n_na
payunsta	My income is likely to be unstable and uncertain	y_n_na
	If you are/were looking for another job, how important would the following aspects be?	
seekcomm	Manageable commuting time	importance5pt_na
seekdist	Distance from the place I live now	importance5pt_na
seekflex	Possibility to have flexitime or bank of hours	importance5pt_na

VARIABLE NAME	SOURCE LABEL	RESPONSE/VALUE
seektrai	Suitable training opportunities	importance5pt_na
seeksala	Decent salary	importance5pt_na
seekperm	Permanent contract	importance5pt_na
seekcoll	Nice co-workers	importance5pt_na
seekfame	Employer's reputation	importance5pt_na
seekpens	Employer contributions to pension	importance5pt_na
seekday1	Employer contributions to child care and health service	importance5pt_na
seekjobq	Quality of job	importance5pt_na
seekhrs	Suitable working hours including possibility of flexitime/ part-time	importance5pt_na
seekstat	Higher designation/social status	importance5pt_na
seektrav	Opportunity to travel abroad	importance5pt_na
seekfrin	Fringe benefits	importance5pt_na
seekrewa	Reward and recognition opportunity	importance5pt_na
seekpar1	Part-time working hours	importance5pt_na
seekcond	Better employment benefits such as higher wage, better wage prospects, other benefits	importance5pt_na
seekintc	International career prospects	importance5pt_na
seekchal	Challenging / interesting job (for example more responsibilities)	importance5pt_na
seekpers	Career prospects	importance5pt_na
seekstres	Less working pressure	importance5pt_na
seekleav	Suitable leave arrangements other than holidays	importance5pt_na
seekcomb	A better fit with family life	importance5pt_na
seekday2	Firm's kindergarten / employer contributions to child-care	importance5pt_na
seekcult	Company culture	importance5pt_na
	More about your working hours	
hrshist1	Have you always worked full-time?	y_n
yyfullti	If no, what year did you start working full-time?	1950_YEAR_NOW
hrshist2	Have you always worked part-time?	y_n
yypartti	If no, what year did you start working part-time?	1950_YEAR_NOW
hrspref	Would you prefer to work more or less hours a week than you usually do?	
	No, I am satisfied with my current hours	0
	Less hours	1
	More hours	2
hrscont4	What sort of working hours have you agreed?	
HINT	If more options apply, please select the one that comes closest	
	Full-time hours per week	1
	Part-time hours per week	2
	Annualised hours	3
	Flexible hours	4
	I work on call	6
	Other	99
	Why do you work part-time?	
hrsptedu	Still in education/training	y_n
hrspthou	Housekeeping	y_n
hrsptchl	Looking after children or other persons	y_n
hrspttim	Time for myself	y_n
hrsptsec	Second job	y_n
hrspthea	Health problems, personal illness or disability	y_n
hrsptret	Early retirement, retirement	y_n
hrsptnof	Cannot find a full-time job	y_n
hrsptdem	Job too stressful or demanding for full-time	y_n
hrsppolt	Policy issues	y_n
hrscont5	How many hours per year do you work under the terms of your contract?	0.15-100.45
hrscont6	Is a minimum or maximum of working hours per week agreed with your employer?	
	A minimum and a maximum	1
	A minimum	2
	A maximum	3
	No	0

VARIABLE NAME	SOURCE LABEL	RESPONSE/VALUE
	Not applicable	-8
hrscont7	How many hours a week do you work under the terms of your contract?	0.15-100.45
wageotim	If you work more hours than laid down in your contract, how is this overtime compensated?	
	Overtime paid as normal hours plus overtime premium	1
	Overtime paid as normal hours	2
	Time-off in lieu for overtime hours	3
	Partly paid, partly compensated with time-off in lieu	4
	Not compensated	5
	I don't know	-7
	Not applicable	-8
hrslunch	Is your lunchtime included in your agreed working hours?	y_n_dk_na
tishift3	The type of shifts or irregular hours you work are	
	Alternating morning / afternoon shifts	2
	Rotating shifts, not including nights and weekends	221
	Rotating shifts, including weekends but no nights	222
	Alternating morning / afternoon / night shifts	3
	Rotating shifts, including nights and weekends	331
	Rotating shifts, including nights but no weekends	332
	Daily split shifts	23
	Morning shifts	9
	Afternoon shifts	8
	Evening shifts	7
	Night shifts	12
	Other schedules	99
	How are your working hours organised?	
tihomtel	I work at home at least one day a week (telework)	y_n_na
tiroste7	I know my working hours at least one month in advance	y_n_na
tiroste8	I know my working hours at best one week in advance	y_n_na
tirost10	My supervisor often asks me to change my working hours	y_n_na
tiroste9	I can easily swap shifts with co-workers	y_n_na
	How often do you find that	
ddrun17	Your supervisor changes your working hours at short notice	neverdaily5pt_na
ticonfli	Your working hours conflict with your family commitments	neverdaily5pt_na
ddrun18	There is no work available for you	neverdaily5pt_na
preftel1	You wish you could work at home now and then (telework)	neverdaily5pt_na
ddrun16	You are unable to take a lunch break	neverdaily5pt_na
ddrun15	You unexpectedly have to work overtime	neverdaily5pt_na
conspeed	You have to work at very high speed	neverdaily5pt_na
tideadli	You have to work to tight deadlines	neverdaily5pt_na
hrschang	Over the 12 past months, have your agreed working hours changed?	
	No	0
	Yes, to less hours	1
	Yes, to more hours	2
	Yes, to flexible hours	3
	Yes, other	4
	Not applicable	-8
hrschan1	If yes, was this	
	On your request	1
	Due to reducing the standard working week with loss of pay	2
	Due to reducing the standard working week without loss of pay	3
	Vanwege deeltijd WW	5
	Due to other reasons	4
ddwork1	In the past seven days, have you worked your usual number of hours?	
	Yes	1
	No, I worked more hours	2
	No, I worked less hours	3
	No, I have not worked at all due to	4
	Why have you worked less hours or not at all?	

VARIABLE NAME	SOURCE LABEL	RESPONSE/VALUE
ddworvac	Vacation / days off	checkbox
ddworill	Illness or injury	checkbox
ddworfam	Family reasons	checkbox
ddworedu	Training course	checkbox
ddworsho	Short-time working	checkbox
ddwordis	Labour dispute / industrial action	checkbox
ddworwea	Weather conditions	checkbox
ddworloo	I was looking for a job	checkbox
ddworlea	Maternity / parental leave	checkbox
ddworsea	Seasonal work	checkbox
ddworsen	Seniority leave	checkbox
ddworvar	Variable working hours / time off in lieu of wages	checkbox
ddworoth	Other reasons	checkbox
confixed	What is the duration of your contract?	checkbox
	1 - 2 days	1
	3 - 6 days	2
	1 - 4 weeks	3
	1 - 3 months	4
	3 - 6 months	5
	0.5 - 1 year	6
	1 - 2 years	7
	2 years or more	8
	Until a task is completed	13
	Not agreed	9
	Not applicable	-8
confixe1	When your contract comes to an end, what are your prospects with your current employer?	
	I expect to become permanently employed	1
	I expect to sign a new fixed-term contract with the same employer	2
	My contract will not be renewed, so I will lose my present job	3
	I don't know	-7
	Other	99
	A fixed-term contract	
confiski	Gives me the opportunity to increase my skills	agree5pt_na
confiexp	Gives me the opportunity to build up work experience	agree5pt_na
confipen	Restricts my capacity to build up a pension	agree5pt_na
confihou	Restricts my ability to buy a house	agree5pt_na
confipar	Forces me to stay with my parents	agree5pt_na
confichi	Forces me to postpone having children	agree5pt_na
confiper	Is the only option because permanent work is not available	agree5pt_na
confiemp	Gives me the opportunity to look for the best employer	agree5pt_na
wageflex	Now we want to ask about your LAST wage. Was your last wage dependent on your individual performance from	
	Piece work	1
	Sales targets	2
	Customers, patients	3
	Agricultural output	4
	Other	99
	None of these	0
wageba1	Did you receive a basic wage?	y_n
wageba2	What was the basic part of your last wage?	amount
	In the past 12 months have you been a participant in the following work related schemes or benefits?	
pastuapp	Apprenticeship	checkbox
pastudys	Education or training scheme	checkbox
papleav1	Maternity / parental leave scheme (above statutory minimum)	checkbox
papleave	Maternity / parental leave scheme	checkbox
padaycre	Company-based day care / creche	checkbox
paleasav	Leave accumulation scheme / time-banking	checkbox

VARIABLE NAME	SOURCE LABEL	RESPONSE/VALUE
pacredit	Preferential credits	checkbox
pachoice	Flexible Benefit Plan	checkbox
pawotime	Working time experiment	checkbox
patelewo	Telework scheme	checkbox
paearlyr	(Partial) early retirement scheme	checkbox
pahrsre2	Arrangement to work shorter hours (such as for working parents)	checkbox
pahrsre1	Statutory entitlement to shorter working hours	checkbox
frpare61	Contribution to parent's day-care	checkbox
frfree61	Additional free days	checkbox
	What do you think about your terms and conditions of employment?	
paypolic	I am satisfied with my employer's approach to pay.	agree5pt_na
payitime	I am confident that I will be paid on time.	agree5pt_na
paycost	I am confident that my wage will keep up with the cost of living.	agree5pt_na
payhilow	The gap between high and low pay is too great at my place of work.	agree5pt_na
paymore	I should earn more, considering my performance.	agree5pt_na
satbonus	I am satisfied with my allowances / bonuses.	agree5pt_na
satcontr	I am satisfied with my kind of contract.	agree5pt_na
pensworr	I am worried about my pension.	agree5pt_na
	More about your household	
hhownho2	Is your home owned or rented?	
	Self-owned without loan	11
	I/We own our home	1
	I/We pay rent	2
	My/Our accommodation is provided rent-free	3
	I/We live in company housing	4
	I/We own our home, but are still paying off the loan	6
	I/We live in the house owned by parents	7
	Institution	12
	Other	99
hhparact	What is your partner's principal activity?	
	Employee on permanent contract	146
	Employee on fixed-term contract	147
	Self-employed, employer, own-account worker, freelance	202
	Paid family worker / working for family business	103
	Working without pay (family business or farm)	306
	Unpaid voluntary worker	307
	Housewife, househusband	308
	Unemployed	410
	Disabled, ill	515
	In education	620
	Retired	916
	In a job creation scheme	130
	In military or comparable services	131
	Other	950
hhhrspa	How many hours a week does your partner usually work?	0.15-100.45
hhincome	What is the usual total net MONTHLY income of your household?	amount
HINT	This is the combined income of all members of the household and may include benefits or other income. Please enter an average if this income varies from month to month.	
	How are household chores divided at home?	
hhrespin	I am the person who contributes most to household income	y_n_na
hhresphh	I am the person who is responsible for most of the household chores	y_n_na
hhhomehe	I/We have paid domestic help	y_n_na
hhshoti1	I generally have enough time for my household chores	y_n_na
hhargume	We often have arguments about the division of household chores	y_n_na
hhtimere	I am trying to cut down the time I spend on household chores	y_n_na
hhwofato	I often find it hard to combine work and family life.	y_n_na
hhgrowfo	Our household grows its own food	y_n_na
hhmutual	We have developed a mutual understanding about sharing household chores	y_n_na

VARIABLE NAME	SOURCE LABEL	RESPONSE/VALUE
hrshkeep	How many hours per week do you spend on housekeeping?	0.15-100.45
hrshelp	How many hours of paid domestic help a week do you have?	0.15-100.45
	Finally, how satisfied are you with	
sathhinc	Your household income	satisfaction5pt_na
sathealt	Your health	satisfaction5pt_na
sathous	Your housing	satisfaction5pt_na
satleisu	The amount of leisure time you have	satisfaction5pt_na
sathhdiv	The division of household chores	satisfaction5pt_na
satwelfa	Welfare provisions	satisfaction5pt_na
	About this questionnaire	
survetxt	If you have any comments on the questionnaire please do so here.	text
prize	Do you want to win the prize?	y_n
nextyear	Are you prepared to complete next year's questionnaire?	y_n

******
